# Impacts of COVID-19 on essential health services in Tigray, Northern Ethiopia: A pre-post study

**DOI:** 10.1371/journal.pone.0256330

**Published:** 2021-08-27

**Authors:** Abraham Aregay Desta, Tewolde Wubayehu Woldearegay, Estifanos Gebremeskel, Mussie Alemayehu, Theodros Getachew, Gebremedhin Gebregzabiher, Kiros Demoz Ghebremedhin, Degnesh Negash Zgita, Abera Berhe Aregawi, Getachew Redae

**Affiliations:** 1 Tigray Health Research Institute, Mekelle, Tigray, Ethiopia; 2 Tigray Regional Health Bureau, Mekelle, Tigray, Ethiopia; 3 Ayder College of Health Sciences, Mekelle University, Mekelle, Tigray, Ethiopia; 4 Ethiopian Public Health Institute, Addis Ababa, Ethiopia; 5 College of Health Sciences, Adigrat University, Adigrat, Tigray, Ethiopia; Purdue University, UNITED STATES

## Abstract

**Background:**

COVID-19 has proved to have an indirect impact on essential health services in several parts of the world which could lead to increased morbidity and mortality and loss of the gains made in the past decades. There were no synthesized scientific evidences which could show the impact of COVID-19 epidemics/pandemic on essential health services in Tigray, Northern Ethiopia. Therefore, this study aimed to assess the impacts of COVID-19 epidemics/pandemic on essential health services provision in Tigray, Northern Ethiopia.

**Methods:**

A pre-post study design was used to assess the impacts of COVID-19 on essential health services delivery in Tigray, Northern Ethiopia in the second quarter of 2020 (Post COVID-19) compared to similar quarter in 2019 (Pre COVID-19). The study focuses on five categories; namely; maternal, neonatal and child health care; communicable diseases with a focus on HIV and TB-HIV co-infection; prevention of mother to child transmission of HIV; basic emergency, outpatient, inpatient and blood bank services, non-communicable diseases and road traffic accidents (RTAs). Analysis was done using Stata version 14.0 software package. The effects of COVID-19 epidemics/pandemic were calculated taking the differences between post COVID -19 and pre COVID-19 periods and the levels of service disruptions presented using proportions. Wilcoxon sign rank test was done and a significance level of ≤0.05 was considered as having significant difference among the two quarters.

**Results:**

There were significant increase in institutional delivery, delivery by Caesarian Section (CS), still birth, postnatal care within 7 days of delivery, the number of children who received all vaccine doses before 1^st^ birthday, the number of under 5 children screened and had moderate acute malnutrition, the number of under 5 children screened and had severe acute malnutrition and children with SAM admitted for management. However, there were significant decrease in HIV testing and detection along with enrolment to antiretroviral therapy (ART) care, number of patients with cardiovascular disease (CVD) risk ≥ 30% received treatment, RTAs, total units of blood received from national blood transfusion service (NBTS) and regional blood banks, total number of units of blood transfused and emergency referral. There were no significant changes in outpatient visits and admissions.

**Conclusion:**

Despite commendable achievements in maintaining several of the essential health services, COVID-19 has led to an increase in under nutrition in under five children, decline in HIV detection and care, CVD, cervical cancer screening and blood bank services. Therefore, governments, local and international agencies need to introduce innovative ways to rapidly expand and deliver services in the context of COVID-19. Moreover, lower income countries have to customize comprehensive and coordinated community-based health care approaches, including outreach and campaigns. In addition, countries should ensure that NCDs are incorporated in their national COVID-19 response plans to provide essential health care services to people living with NCDs and HIV or HIV-TB co-infection during the COVID-19 pandemic period.

## Introduction

Worldwide, different outbreaks have affected the social and economic wellbeing of communities [1]. Outbreaks do have direct and indirect effects. While health systems are overwhelmed by direct mortality, indirect mortality from vaccine-preventable and treatable conditions increases dramatically [2]. For instance, in 2014, an analysis from the Ebola virus outbreak in West Africa showed that the effects of the Ebola outbreak were extremely less than the indirect effects of the outbreak which is an indication of the need to respond to both the immediate and indirect effects of a pandemic [1].

Similarly, the pandemic of a novel Coronavirus Disease 2019 (COVID-19) which is caused by severe acute respiratory syndrome coronavirus 2 (SARS-CoV2) has posed a severe global crisis [3]. Health systems around the world regardless of income level are struggling to curb the rapid spread of COVID-19 [4]. In an effort, the international community is mobilizing to limit the spread of severe acute respiratory syndrome coronavirus 2 and reduce mortality from COVID-19 [5]. Following that, many countries are responding to COVID-19 pandemic through national or local lockdowns to halt the spread of the disease. While the world’s attention is understandably focused on the direct impact of the COVID-19 pandemic, it is essential to see the health crisis from a broader perspective [6].

Evidence indicates that the COVID-19 pandemic has made significant disruption in health service delivery particularly in resource-limited countries [7]. The health disruption is not only due to the direct effects of the COVID-19 pandemic but also it pressurized the health systems and stretched others beyond their capability indirectly. COVID-19 is reported to have a spotlight on revealing holes in the healthcare delivery system that can have lasting side effects on patients and providers [8]. Failure to protect health care exposes the health systems for critical gaps in service delivery when they are most needed and can have a long-lasting impact on the health and wellbeing of population [9]. However, the COVID-19 pandemic begins disrupting preventive and treatment services for non-communicable diseases (NCDs) and it known that people living with NCDs are at higher risk of severe COVID-19 related illness and death [10].

The COVID-19 pandemic is causing widespread disease and death. In addition to the direct cause, the pandemic poses a significant risk of indirect morbidity and mortality from other preventable and treatable diseases as a result of essential health services disruption [11]. The most common reasons mentioned for critical gaps or reducing services during COVID-19 were cancellations of planned treatments, decrease in public transport and repurposing of health care workers to support COVID-19 services [10]. Besides, loss of income due to lockdowns may limit people’s ability to pay for services and limit utilization [12–14] and high rates of morbidity and mortality among health care workers, were another reason leading to staff shortages [15]. One in five countries have reported shortage of medicines, diagnostics and other technologies as the main reasons for disruption of services [10].

Modeling estimates using the lives saved tool (LiST) shows that COVID-19-related disruptions could leave many women and children without access to essential services and result in an increased maternal and child morbidity and mortality [16]. A survey in some healthcare facilities in Tigray region has showed that the effect of COVID-19 has pressurized the provision of essential health services, from a reduction in the number of mothers coming for antenatal care to the number of women delivering their babies in health facilities [17]. Lockdown in movement and the reluctance of parents or caregivers to visit the health facilities being fearful of contracting the coronavirus were the reasons identified [17]. This may further affect the health system being burdened not only by the pandemic but also by people seeking health care when it is too late. Therefore, minimizing the impact of this public health emergency successfully requires every health resource to be leveraged [9].

Despite a rapid increase in the number of COVID-19 cases and deaths in Ethiopia, there is limited synthesized data on the indirect impacts of COVID-19 and the reasons for service disruption. Similarly, there were no synthesized scientific evidence which show the indirect impacts of COVID-19 epidemics/pandemic on essential health services in Tigray, Northern Ethiopia. Therefore, this study aimed to assess the indirect impacts of COVID-19 epidemics/pandemic on essential health services in Tigray, Northern Ethiopia. This study can represent the Ethiopian context and it could help policy makers to effectively manage the indirect effects of COVID-19 in the health care system of Ethiopia and other similar low-and middle income countries (LMICs).

## Methods

### Study setting and period

**Fig 1:**
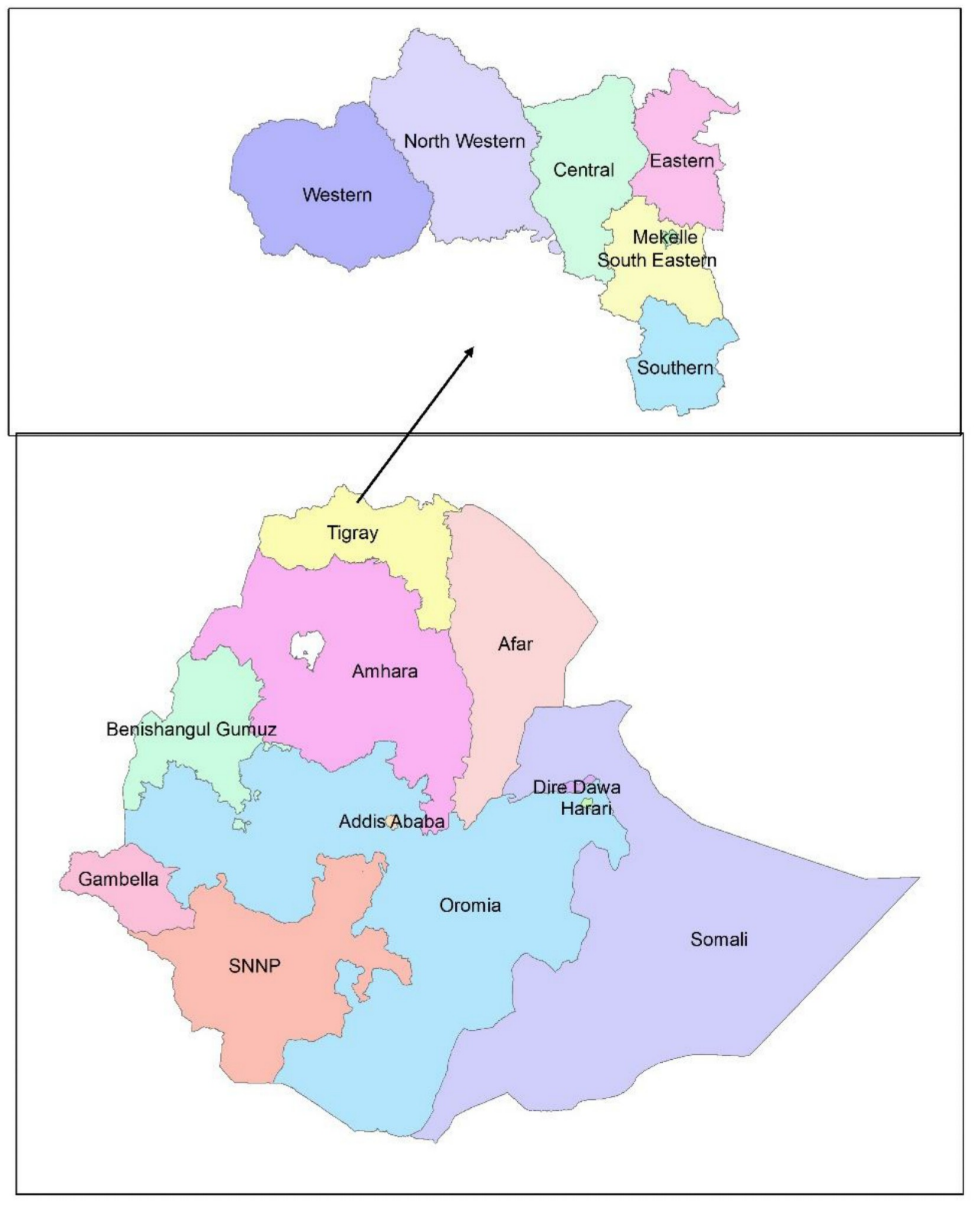
Study area: To show where the region is located (sourced self).

This study was conducted in Tigray region, Northern Ethiopia (Fig 1). Tigray region is the 4^th^ most populous and the 6^th^ largest by surface area among the 9 regional states of Ethiopia. The region had 52 districts, among which 18 are urban districts. The region had an estimated population of 5,055,999 in 2016. Health care services are provided through both public and private health care facilities. There are 2 specialized referral hospitals, 16 public general hospitals, 22 public primary hospitals, 202 public health centers, and 712 public health posts in the region. Additionally, there are more than 500 private health care facilities including two general hospitals [18].

### Study design and participants

A pre-post study design was used to assess the impacts of COVID-19 on essential health services delivery in Tigray, Northern Ethiopia during the second quarter of 2020 (Post COVID-19) compared to similar quarter in 2019 (Pre COVID-19) (3 months period). This helps to assess the effects of COVID-19 on essential health services delivery by comparing with similar period before the occurrence of COVID-19.

### Eligibility criteria

All health care facilities that reported using DHIS-2 under the jurisdiction of the regional health bureau were included in the study.

#### Sampling

The study used a purposive sampling technique.

### Data sources and management

The source of the data was the Demographic Health Information System-2 (DHIS-2) database of the region. Components of the DHIS-2 variables reported to the region by the district health offices, general hospitals and referral hospitals were directly measured as a count data in this study. Monthly data from the DHIS-2 were exported to Microsoft excel 2013. Basic indicators from each of the essential health service categories were selected and extracted from the Microsoft excel 2013 and exported to Stata 14.0 for analysis (S1 File).

### Definitions and measurements

**ANC 4 visit**: Number of pregnant women that received four or more antenatal care visits.

**DPT1-HepB1-Hib1 (pentavalent First dose) immunization coverage (< 1 year)**: Proportion of surviving infants who have received first (one) dose of the combined diphtheria, tetanus toxoid, pertussis, Hepatitis B & Haemophiles influenza type B vaccine.

**Essential health services**: An Essential Health Services is defined as the package of services that the government is providing or aspiring to provide to its citizens in an equitable manner.

**Family planning acceptance rate**: Proportion of women of reproductive age (15–49 years) who are not pregnant and are accepting a modern contraceptive method (new and repeat acceptors).

**Inpatient mortality rate**: Inpatient deaths before discharge per 100 patients discharged **Institutional neonatal mortality rate**: number of neonates who died before discharge per 1000 livebirths.

**Institutional stillbirth rate**: number of babies born in the institution with no signs of life, with a gestational age of 28 weeks or more, per 1000 (still and live) births.

**Level of service disruption/gain**: The level of decrease or increase in essential health services delivery during COVID-19 pandemic in the second quarter of 2020 compared with similar quarter in 2019.

**Institutional maternal deaths**: Number of maternal deaths in health facility.

**Measles first dose immunization (MCV1) coverage (< 1 year)**: Proportion of surviving infants who have received first dose measles (MCV1) vaccine before their first birthday.

**Measles second dose (MCV 2) Immunization coverage (12–24 months)**: Proportion of children who have received a second dose of measles vaccine before their second birthday.

**Number of patients with CVD risk > = 30% received treatment**: Number of suspected patients who meet 30% of the clinical assessments for CVD prediction started treatment.

**PITC**: Provider initiated HIV testing and counseling (PITC) is HIV testing initiated by a health worker.

**PMTCT**: A program offered in a range of services for reproductive age women living with or at risk of HIV to maintain their health and halt transmission of HIV to their infants.

**Post COVID-19**: Refers to the second quarter of 2020 after the occurrence of COVID-19.

**Pre COVID-19**: Refers to the second quarter of 2019 before the occurrence of COVID-19.

**Second quarter**: Extends from April 29 to June 27 which coincides with the timing of reporting of DHIS-2 data in Ethiopia.

**Skilled delivery**: The number of births attended by skilled health personnel at a health facility.

**VCT**: It is the process by which an individual undergoes counseling, enabling him or her to make an informed choice about being tested for HIV.

### Data analysis

The findings of the study are presented in five categories; namely, maternal, neonatal and child health care; communicable diseases with a focus on HIV and TB-HIV co-infection; prevention of mother to child transmission of HIV; basic emergency, outpatient, inpatient and blood bank services and non-communicable diseases and road traffic accidents (RTAs).

Data were analyzed using Stata version 14.0 statistical software package. The analysis was conducted among the 52 districts, 14 public and 2 private general hospitals, and 2 comprehensive specialized hospitals. To assess the effect of the COVID-19, the regional sum of performance before COVID-19 was subtracted from a similar period of 2020. The percentage of reduction or increase was calculated by “post COVID-19 minus pre COVID-19 divided by pre COVID-19 performance and finally multiplied by 100”. Variables with a discrete data were checked for normality using the Shapiro-Wilk normality test. Variables with a significant p-value ≤0.05 in the Shapiro-Wilk normality test were considered as non-normally distributed observations. For those with non-normally distributed variables, a median was used to compare the difference between pre and post COVID-19 pandemic. Wilcoxon sign rank test was done among the 70 paired observations which compared the 2020 and 2019 second quarter performances using the indicators on maternal, neonatal and child health, HIV and TB/HIV co-infection, Prevention of mother to child transmission of HIV (PMTCT) services, emergency and gross indicators for outpatient and inpatient services during the COVID-19 pandemic with similar quarter performance in 2019. Wilcoxon sign rank test was determined at α level 0.05 to check whether the median difference between the similar periods was statistically significantly different from zero. Significance level of ≤0.05 was considered as having difference between the two quarters.

## Results

### Effects of COVID-19 pandemic on maternal, neonatal and child health care services

The analysis of the study showed that there was a decrease of 4.81%, 2.83%,12.30%, 0.21%, 4.13%, in family planning new acceptors, ANC 4 visits, number of women who received comprehensive abortion care, number of children under two years of age who have received second dose of measles, and number of children treated for diarrhea with ORS and zinc respectively.

There were an increase in number of still births (18.57%) and still birth rate (7.64%), number of children <5 year screened and have severe acute malnutrition (30.99%) and death among children admitted for SAM (117.86%). On the other hand, there was an increase in skilled delivery attendance (8.57%), postnatal care within 7 days of delivery (13.99%), delivery by caesarian section (CS) (28.05%), pentavalent first dose coverage (14.86%), number of children who received all vaccine doses before 1^st^ birthday (14.71%) and measles second dose (MCV 2) Immunization coverage (12–24 months) (15.51%) (Table 1).

**Table 1:** Effects of COVID-19 on maternal, child and neonatal health care services in Tigray, Northern Ethiopia, 2020.

Type of service	Indicator	Sum Pre COVID-19	Sum Post COVID-19	Difference (Post COVID-19 minus Pre COVID-19)	Level of service disruption or gain (%)	Wilcoxon signed rank test P-value
Maternal	Family Planning new acceptors	39526	37623	-1903	-4.81%	0.0372
	Family planning acceptance rate	53.67%	55.53%	1.86%	3.47%	0.1569
	Family planning repeat acceptors	106298	112780	6482	6.10%	0.0217
	ANC at least once	47896	50327	2431	5.08%	0.0978
	ANC 4 times	30523	29658	-865	-2.83%	0.5761
	Skilled delivery	33888	36792	2904	8.57%	0.0001
	Still births	560	664	104	18.57%	0.0062
	Institutional maternal deaths	23	19	-4	-17.39%	0.3173
	Postnatal care within 7 days of delivery	38305	43662	5357	13.99%	0.0003
	Delivery by caesarian section (CS)	2392	3063	671	28.05%	0.0040
	Number of women received comprehensive abortion care	5950	5218	-732	-12.30%	0.3133
	Still birth rate	15.7	16.9	1.2	7.64%	0.0618
Neonates	Number of neonatal deaths in the first 7 days of life (institutional)	784	417	-367	-46.81%	0.0733
Children	DPT1-HepB1-Hib1 (pentavalent First dose) immunization coverage (< 1 year)	98.01%	112.57%	14.56%	14.86%	<0.0001
	Measles first dose immunization (MCV1) coverage (< 1 year)	90.01%	103.67%	13.66%	15.18%	<0.0001
	Number of children received all vaccine doses before 1^st^ birthday	35763	41024	5261	14.71%	<0.0001
	Measles second dose (MCV 2) Immunization coverage (12–24 months)	49%	56.6%	7.6%	15.51%	0.1313
	Number of children under two years of age who have received second dose of measles	25866	25812	-54	-0.21%	0. 3800
	Number of children treated for diarrhea with ORS and zinc	36337	34838	-1499	-4.13%	0.1679
	Number of children less than 2 years weighted during GMP session	211729	215393	3664	1.73%	0.2316
	Number of weights recorded with moderate malnutrition (Z score between -2 and -3)	19323 (9.13%)	23604 (10.96)	4281	22.15%	0.5606
	Number of weights recorded with severe malnutrition (Z score below -3)	3650 (1.72%)	3232 (1.50%)	-418	-11.45%	0.0929
	Total number of children <5 years screened for acute malnutrition	1178916	1196194	17278	1.47%	0.6012
	Number of children <5 year screened and have moderate acute malnutrition	42762 (3.63%)	54621 (4.57%)	11859 (0.94%)	27.73%	0.0069
	Number of children <5 year screened and have severe acute malnutrition	3753 (0.32%)	4916 (0.41%)	1163 (0.09%)	30.99%	0.0002
	Children with SAM admitted for management	1405	3061	1656	117.86%	<0.0001
	Death among children admitted by SAM	3	44	41	1366.67%	0.1797
	Death rate among the admitted SAM under 5 years children	0.21%	1.44%	1.23%	585.71%	0.6547
	Total number of children aged 6–59 months who received Vitamin A supplementation by age	263183	267978	4795	1.82%	0.3035

Wilcoxon signed rank test has showed that there was a significant increase in skilled delivery (p-value = 0.0001), postnatal care within 7 days of delivery (p-value = 0.0003), delivery by caesarian section (CS p-value = 0.0040) and number of children who received all vaccine doses before 1^st^ birthday (p-value = <0.0001). Similarly, the Wilcoxon signed rank test has showed a significant increase in the number of children <5 year screened and have moderate acute malnutrition (p-value = 0.0069), number of children <5 year screened and have severe acute malnutrition (p-value = 0.0002) and number of children admitted for SAM management (p-value = <0.0001) (Table 1).

### Effects of COVID-19 pandemic on HIV and TB/HIV co-infection services

The analysis clearly showed that there was a decrease in all indicators except the following indicators where an increase was observed: number of people living with HIV (PLHIV) who were screened and found to be clinically undernourished (60.31%), number of PLHIV that were nutritionally assessed and found to have MAM (% MAM among screened) (58.58%), and number of PLHIV who were nutritionally assessed and found to have SAM (% of SAM among screened) (40.05%). Wilcoxon signed rank test has showed a significant decrease in voluntary testing and counseling (VCT) testing (p-value = <0.0001) and yield of positivity in VCT (p-value = 0.0051). However, the positivity rate at VCT (p-value = 0.1662) compared to the comparison period was not statically significant despite its reduction in absolute numbers. Similarly, it was shown that there was a decrease in the number of individuals tested in PTIC (p-value = 0.0158), HIV positive yield at PTIC (p-value = <0.0001), positivity rate at PTIC (p-value = 0.0019), total number of individuals tested for HIV (p-value = 0.0006), total yield of HIV positivity (p-value = 0.0026) and total HIV positivity rate in all HIV testing sites (p-value = 0.0045). Likewise, there was a statistically significant decrease in number of adults and children with HIV infection newly started on ART (p-value = 0.0001) and number of clients enrolled in HIV care who were screened for TB (p-value = 0.0318) (Table 2).

**Table 2:** Effects of COVID-19 on HIV and HIV-TB co-infection in Tigray, Northern Ethiopia, 2020.

Type of service	Indicator	Pre COVID-19	Post COVID-19	Difference (Post COVID-19 minus Pre COVID-19)	Level of service disruption or gain (%)	Wilcoxon signed rank test P-value
HIV and TB-HIV co-infection	Individuals tested at VCT	41024	24712	-16312	-39.76%	<0.0001
	Positive yield at VCT	358	186	-172	-48.04%	0.0051
	Positivity rate at VCT	0.26%	0.17%	-0.09%	-34.62%	0.1662
	Individuals tested at PITC	96060	84665	-11395	-11.86%	0.0158
	Positive yield at PITC	780	207	-573	-73.46%	<0.0001
	Positivity rate at PTIC	0.81%	0.24%	-0.57	-70.37%	0.0019
	Total number of individuals tested for HIV	137084	107130	-29954	-21.85%	0.0006
	Total yield of HIV positivity	1138	393	-745	-65.47%	0.0026
	Total HIV positivity rate in all site of tests	0.49%	0.20%	-0.29	-59.18%	0.0045
	Number of adults and children with HIV infection newly started on ART	671	360	-311	-46.35%	0.0001
	Number of PLHIV who were screened for malnutrition	64274	62093	-2181	-3.39%	0.6089
	Number of PLHIV who were screened and found to be clinically undernourished	5633	9030	3397	60.31%	0.1544
	Number of PLHIV that were nutritionally assessed and found to have MAM (% MAM among screened)	3037 (4.73%)	4816 (7.76%)	1779 (3.03%)	58.58%	0.0658
	Number of PLHIV that were nutritionally assessed and found to be SAM (% of SAM among screened)	1628 (2.53%)	2280 (3.67%)	652 (1.14%)	40.05%	0.1527
	Number of clients enrolled in HIV care who were screened for TB	84158	65042	-19116	-22.71%	0.0318
	Total number of HIV positive clients identified with active TB	128	56	-72	-56.25%	0.5598
	% active TB among screened	0.15%	0.09%	-0.06	-40.00%	0.4236

### Effects of COVID-19 on prevention of mother to child transmission

The total number of new women who received ART and number of HIV exposed infants who received ARV prophylaxis for 12 weeks have increased during the COVID-19 epidemics/pandemic period (976.47%). However, there were reductions in the number of new HIV positive women during ANC, L&D and postpartum (-38.37%); infants virologically positive within 12 months (-10.00%); number of HIV exposed infants who received ARV prophylaxis for 6 weeks (-14.93%) and number of partners of pregnant, laboring and lactating women whose test is positive for HIV (-68.06%). The Wilcoxon signed rank test has showed that there were no significant differences pre COVID-19 and post COVID-19 pandemic (Table 3).

**Table 3:** Effect of COVID-19 on prevention of mother to child transmission of HIV, Tigray, Northern Ethiopia, 2020.

Type of service	Indicator	Pre COVID-19	Post COVID-19	Difference (Post COVID-19 minus Pre COVID-19)	Level of service disruption or gain (%)	Wilcoxon signed rank test P-value
	Number of women tested in ANC, Labor and delivery and PNC	43199	42072	-1127	-2.61%	0.4076
	Number of new HIV positive women during ANC, L&D and Postpartum	86	53	-33	-38.37%	0.3489
	Total new number of women received ART	186	328	142	76.34%	0.4203
	Total number of infants within 12 months received virological test result	199	202	3	1.51%	0.6083
	Infant virologically positive within 12 months	10	9	-1	-10.00%	0.5562
	Number of HIV exposed infants who received ARV prophylaxis For 6 weeks	268	228	-40	-14.93%	0.6341
	Number of HIV exposed infants who received ARV prophylaxis For 12 weeks	17	183	166	976.47%	0.1128
	Number of partners of pregnant, laboring and lactating women tested for HIV and know their results	8029	8177	148	1.84%	0.8033
	Number of partners of pregnant, laboring and lactating women whose test result is HIV positive	72	23	-49	-68.06%	0.6547
	Number of HIV exposed infants receiving HIV confirmatory (antibody test) by 18 months	264	245	-19	-7.20%	0.1709
	Number of confirmed HIV positives among tested exposed infants receiving HIV confirmatory (antibody test) by 18 months	5	4	-1	-20.00%	-^A^

^A^ = unadjusted variance, adjustment for ties, adjustment for zeros and adjusted variance were zero and hence unable to calculate the p-value

### Effects of COVID-19 pandemic on non-communicable diseases and road traffic accidents (RTAs)

As shown in Table 4, the number of women 30 to 49 years of age who were screened for cervical cancer using visual inspection with acetic acid (VIA) decreased by -54.82% during the epidemics/pandemic period. But the Wilcoxon signed rank test has shown that there was no statistical difference between the two quarters (p-value = 0.1540). On the other hand, the Wilcoxon signed rank test has shown that there was a statistically significant difference in number of patients with Cardiovascular disease (CVD) with the risk of > = 30% who received treatment (p-value = 0.03173) and RTAs (p-value = 0.0465) (Table 4).

**Table 4:** The effects of COVID-19 on non-communicable diseases and RTAs in Tigray, Northern Ethiopia, 2020.

Type of service	Indicator	Pre COVID-19	Post COVID-19	Difference (Post COVID-19 minus Pre COVID-19)	Level of service disruption or gain (%)	Wilcoxon signed rank test P-value
Cervical cancer	Number of women 30 to 49 years of age screened for cervical cancer using VIA	695	314	-381	-54.82%	0.1540
	Normal cervix	605	299	-306	-50.58%	0.1386
	Detected Precancerous lesion from the VIA	32	6	-26	-81.25%	0.1655
	Cancerous lesion	57	9	-48	-84.21%	0.0897
	Number of women aged 30–49 years with cervical lesion received treatment	20	3	-17	-85.00%	0.3173
CVD	Number of patients with CVD risk > = 30% received treatment	1238	15	-1223	-98.79%	0.03173
Road Traffic accidents (RTAs)	Road traffic accidents	1634	1050	-584	-35.74%	0.0465

### Basic emergency, outpatient, inpatient and blood bank services

Although the number of admissions and discharges decreased by -9.95% and -17.78% respectively during the COVID-19 pandemic, there was higher number of deaths (4.24%) during the time of COVID-19 pandemic. However, the total number of emergency unit attendances and emergency referrals had increased by 9.90% and 18.94% respectively.

On the other hand, the total units of blood received from NBTS and regional blood banks, the total number of units of blood transfused, emergency mortality rate and the number of non-emergency referrals have decreased by -7.86%, -13.69%, -77.03% and -26.97% respectively during the pandemic period. Despite these reductions, only the total units of blood received from NBTS and regional blood banks (p-value = 0.0084), the total number of units of blood transfused (p-value = 0.0401) and emergency referrals (p-value = 0.0076) were the only ones that had statistically significant difference using Wilcoxon signed rank test (Table 5).

**Table 5:** The effects of COVID-19 outbreak on basic emergency, outpatient, inpatient and blood bank services, Tigray, Northern Ethiopia, 2020.

Type of service	Indicator	Pre COVID-19	Post COVID-19	Difference (Post COVID-19 minus Pre COVID-19)	Level of service disruption or gain	Wilcoxon signed rank test P-value
Outpatient	OPD attendances	2608402	2637896	29494	1.13%	0.8206
Inpatient	Admission	30806	27740	-3066	-9.95%	0.2575
	Bed Occupancy Rate (BOR)	48.4	40.8	-7.6	-15.70%	0.6339
	Discharged	30527	25098	-5429	-17.78%	0.0875
	Inpatient death	708	738	30	4.24%	0.7365
Blood bank services	Total units of blood received from NBTS and regional blood banks	3066	2825	-241	-7.86%	0.0084
	Total number of units of blood transfused	3433	2963	-470	-13.69%	0.0401
Emergency	Total number of emergency unit attendances	123144	135337	12193	9.90%	0.2607
	Total deaths in the emergency unit	913	226	-687	-75.25%	0.8469
	Emergency mortality rate	0.74	0.17	-0.57	-77.03%	0.3221
	Emergency referral	7288	8668	1380	18.94%	0.0076
	Non-emergency referral	18196	13289	-4907	-26.97%	0.4364

**Fig 2:**
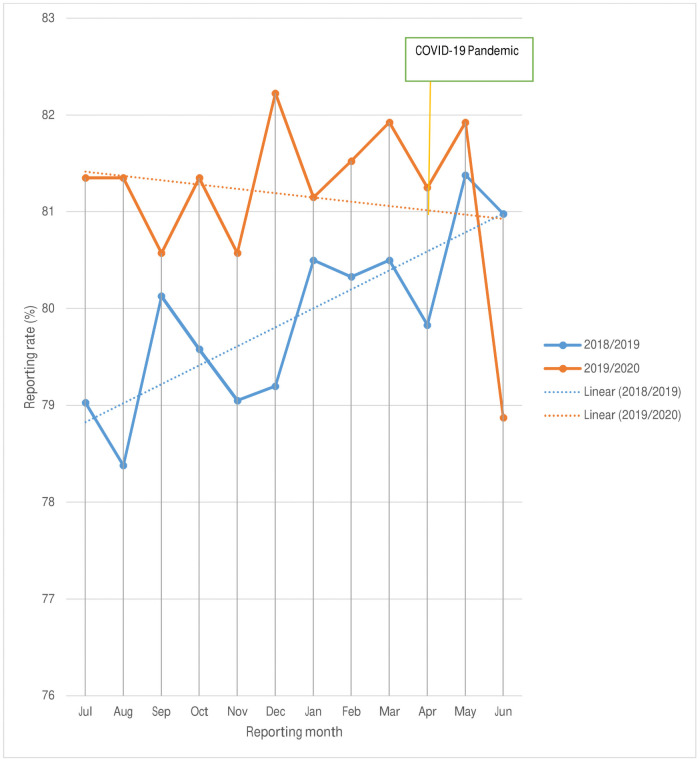
Reporting status of health care facilities in 2018/2019 and 2019/2020.

The average DHIS-2 reporting rate of the health care facilities had improved in 2019/2020 as compared to the 2018/2019 period as shown below. The rate of reporting in June 2020 was reduced by 2.11% compared to June 2019. Even though the line graph has shown a better reporting rate in 2019/2020, the linear trend line was decreasing across the months (Fig 2). However, there were no difference in the reporting rates (p- value = 1.000) among the two quarters of 2019 and 2020.

## Discussion

The main aim of the study was to assess the impacts of COVID-19 on essential health services in Tigray, Northern Ethiopia. The study found that there was a significant increase in skilled delivery, postnatal care within 7 days of delivery, delivery by caesarian section (CS), number of children who received all vaccine doses before 1^st^ birthday. Similarly, there was a significant increase in number of still births, number of children <5 year screened and have severe acute malnutrition and number of children admitted for SAM management. Indicators on essential health services in HIV and HIV-TB co-infection have showed a significant decrease in testing and yield of positivity, enrolment to ART care and TB screening. Similarly, there was a decrease in number of women aged 30–49 years with cervical lesion and received treatment. The study has also showed that there was a statistically significant median difference in number of patients with CVD risk > = 30% who received treatment and road traffic accidents. There was a decrease in the number of admissions, total units of blood received from NBTS and regional blood banks, emergency mortality rate and number of non-emergency referrals. But, the number of deaths in the inpatient have increased during COVID-19 pandemic.

The study found that there was a significant increase in skilled delivery, postnatal care within 7 days of delivery, delivery by caesarian section (CS), number of children received all vaccine doses before 1^st^ birthday. Similar study conducted in Dessie referral hospital, Ethiopia showed that despite the fact that different services decreased during the COVID-19 pandemic period, the number of mothers delivering at Dessie referral hospital remained relatively stable [19]. This study has showed an increase in first antenatal but decrease in ANC 4 times visit. But a study in Addis Ababa and Dessie referral hospital has showed a decrease in ANC 1^st^ visit by 12% and 50% respectively during the COVID-19 pandemic [19, 20]. The variations could be attributed due to differences in time periods and source of the data used.

There was a significant increase in number of still births, number of children <5 year screened and have moderate acute malnutrition, number of children <5 year screened and have severe acute malnutrition and number of children admitted for SAM management. A study from Nepal has reported that there was a sharpest increase in the number of still births in the first four weeks of the COVID-19 pandemic and lockdown [21]. This might be justified that a pregnant women might have received less care than they need because of the lockdown restrictions and disruptions to health care. As a result, complications that can lead to stillbirths had probably increased.

On the other hand, there is report that the COVID-19 pandemic is undermining nutritional status of communities across the world, particularly in low-income and middle-income countries (LMICs) [22]. Other study has showed that child malnutrition, including wasting is expected to increase. This is due to steep declines in household incomes, changes in the availability and affordability of nutritious foods, and interruptions to health, nutrition, and social protection services [23]. According to the International Food Policy Research Institute, economic, food, and health systems disruptions resulting from the COVID-19 pandemic are expected to continue to exacerbate all forms of malnutrition [24]. There are three underlying causes of malnutrition during COVID-19 pandemic: (a). household food insecurity due to loss of income particularly among the lower wealth quintile households with children under 5; (b). Caring practices for children are likely to go down as livelihoods are affected; and (c). Access to health services for common child illnesses and treatment of moderate and severe wasting maybe be disrupted due to health workers’ limited access to health facilities or shifted to COVID-19 responses [25]. Overall the main reason for having malnutrition might be due to the strategies to respond to COVID-19 such as physical distancing, school closures, trade restrictions, and country lockdowns might have impacted on food systems by disrupting the production, transportation, and sale of nutritious, fresh, and affordable foods, that have forced millions of families to rely on nutrient poor alternatives [26].

Analysis of essential health services on HIV and HIV-TB co-infection have showed a significant decrease in testing and yield of positivity in VCT, number of individuals tested and HIV positivity yield at PTIC, positivity rate at PTIC, total number of individuals tested for HIV, total yield of HIV positivity and total HIV positivity rate in all HIV testing sites, number of adults and children with HIV infection newly started on ART and number of clients enrolled in HIV care who were screened for TB. In low and middle income countries, a particular concern is the potential impact on HIV and tuberculosis, due to a possible disruption to health services. COVID-19 is already affecting control measures for tuberculosis and HIV [27]. This may be due to fear of COVID-19 transmission during the health facility visit.

There was a decrease in number of women aged 30–49 years with cervical lesion and received treatment in the pandemic period compared to the similar period which was used as a comparison. The study has also showed that there was a statistically significant median difference in number of patients with CVD risk > = 30% who received treatment and road traffic accidents. A study from Dessie referral hospital, Ethiopia has shown that the number of patients coming for medical chronic illness follow-up decreased [19]. According to the Pan American Health Organization (PAHO) and WHO, services for the prevention and treatment of non-communicable diseases (NCDs) have been critically affected since the onset of the COVID-19 pandemic in the region of the Americas. When the pandemic began, routine health services were reorganized or interrupted and many stopped providing care to people in treatment for cancer, cardiovascular diseases, and diabetes, among others. Many of the health workers who usually provide this kind of care were reassigned to the COVID-19 response. Outpatient services for non-communicable diseases were interrupted in many countries. These disruptions have affected all types of care for people with NCDs, but more so for diabetes, hypertension, dental care, and rehabilitation services. The main reasons cited for disruption of NCD services include cancellation of elective care services, clinical staff being reassigned to COVID-19 response, patients not presenting, postponement of screening programs, cancellation of scheduled treatments, reduced availability of public transportation, fear of visiting health care centers, and staff shortages due to reassignments to support the COVID-19 response, shortage of medicines, diagnostics and other technologies. Also, some countries experienced disruptions in their supply chains and faced challenges in the distribution of drugs and health products, all of which has affected patients’ access to services [28].

There were significant reduction RTAs in this study, which is similar with other findings. Evidences from Spain has revealed the disruptive effect of the COVID-19 pandemic on transportation and road safety. During the COVID-19 lockdown in Tarragona province, overall mobility declined to 62.9%, while traffic accidents fell to 74.3% [29]. Similarly, in Missouri State, USA there was a significant reduction in road traffic accidents resulting in minor or no injuries but not in accidents resulting in serious or fatal injuries after mandated societal lockdown [30]. This is because a minimized mobility, which leads a lesser probability of crashes.

There was a decrease in the number of admissions, discharges, total units of blood received from NBTS and regional blood banks, total number of units of blood transfused, total number of deaths in the emergency unit, emergency mortality rate and number of non-emergency referrals were decreased during the COVID-19 pandemic period and lockdown. But, the number of deaths in the inpatient care have increased. Despite decreases, only total units of blood received from NBTS and regional blood banks, total number of units of blood transfused and emergency referral have had statistically significant differences between the pandemic period and comparison period. National Syndromic Surveillance Program (NSSP) found that emergency department (ED) visits declined by 42% during the early COVID-19 pandemic in United States of America [31]. A study in Zambia showed a decrease in the number of blood units collected for transfusion [32]. Similarly, a study from India reported that blood/ component shortage, convalescent plasma, consumable logistics and supply and donor and staff safety were the challenges in COVID-19 pandemic [33]. In the COVID-19 pandemic period in Saudi Arabia, donor attendance and blood supply at blood bank-based collections and blood demand have reduced by 39.5% and 21.7% respectively [34]. This because of lockdown which inhibits volunteers from providing blood donation and recipients from health care visits.

In summary, there were remarkable decline in HIV detection and care; blood bank services, RTAs, inpatients admissions; CVD and Cervical cancer screening and detection. On the other hand, there were a significant increase in still birth, undernutrition among children less than 5 years old and inpatient mortality. Although, there were a significant increase in delivery and CS and immunization services, there were no changes in outpatient and inpatient visits. The significant reduction might be due to fear of movement, lockdown, health care workers re-assigned to COVID-19 response and reduction of income.

### Strength and limitations of the study

The study was not without limitation. The main limitation of this study was that the analysis was not at individual level. Likewise, the reporting quality may affect the findings of the study. Despite these limitations, the study had strengths: it was done across all key programs which can clearly show the disruption of essential health services delivery in the region. Moreover, the impacts of COVID-19 in the second quarter of 2020 were determined by comparing with similar quarter in 2019 which controls for seasonality.

## Conclusion

Despite commendable achievements in maintaining several of the essential health services, COVID-19 has led to increment in under nutrition in under five children, decreased HIV detection and care, chronic vascular disease, cervical cancer screening and blood bank services. Therefore, governments, local and international agencies need to introduce innovative ways to rapidly expand and deliver services in the context of COVID-19. Moreover, low income countries have to customize comprehensive and coordinated community based health care approaches, including outreach and campaigns. In addition, countries should ensure that NCDs are incorporated in their national COVID-19 response plans to provide essential health care services to people living with NCDs and HIV or HIV-TB co-infection during the COVID pandemic period.

## Supporting information

S1 File This is the dataset. (XLSX)
